# YAP-mediated trophoblast dysfunction: the common pathway underlying pregnancy complications

**DOI:** 10.1186/s12964-023-01371-2

**Published:** 2023-12-14

**Authors:** Qimei Lin, Jiasong Cao, Jing Yu, Yu Zhu, Yongmei Shen, Shuqi Wang, Yixin Wang, Zhen Liu, Ying Chang

**Affiliations:** 1 Tianjin Key Laboratory of Human Development and Reproductive Regulation, Nankai University Affiliated Maternity Hospital, Tianjin Central Hospital of Obstetrics and Gynecology, Tianjin, 300100 China; 2 School of Clinical Medicine, Tianjin Medical University, Tianjin, 300070 China; 3 School of Medicine, Nankai University, Tianjin, 300071 China; 4 Present Address: Academy of Clinical Medicine, Medical College, Tianjin University, Tianjin, 300072 China

**Keywords:** YAP, Trophoblast, Pregnancy complications, Maternal–fetal interface, Placental dysfunction, Inflammation

## Abstract

Yes-associated protein (YAP) is a pivotal regulator in cellular proliferation, survival, differentiation, and migration, with significant roles in embryonic development, tissue repair, and tumorigenesis. At the maternal–fetal interface, emerging evidence underscores the importance of precisely regulated YAP activity in ensuring successful pregnancy initiation and progression. However, despite the established association between YAP dysregulation and adverse pregnancy outcomes, insights into the impact of aberrant YAP levels in fetal-derived, particularly trophoblast cells, and the ensuing dysfunction at the maternal–fetal interface remain limited. This review comprehensively examines YAP expression and its regulatory mechanisms in trophoblast cells throughout pregnancy. We emphasize its integral role in placental development and maternal–fetal interactions and delve into the correlations between YAP dysregulation and pregnancy complications. A nuanced understanding of YAP's functions during pregnancy could illuminate intricate molecular mechanisms and pave the way for innovative prevention and treatment strategies for pregnancy complications.

Video Abstract

**Supplementary Information:**

The online version contains supplementary material available at 10.1186/s12964-023-01371-2.

## Background

The Yes-associated protein 1 (YAP1), hereafter referred to as YAP, is a pivotal effector of the Hippo signaling pathway. It is renowned for its essential roles in embryonic development, organ regeneration, and tissue homeostasis [1–3]. The YAP signaling dysregulation is associated with the onset of various diseases [4–6]. Recent studies have revealed that YAP can sense intrinsic biochemical and extrinsic biomechanical cues, including mechanical signals, cell polarity, and energy status, and convert them into soluble cellular signals and metabolic pathways, which are instrumental in determining cell fate [7]. This intricate process is meticulously regulated, with the phosphorylation of YAP protein being a cardinal step. Phosphorylated YAP is sequestered in the cytoplasm and degraded by the ubiquitin–proteasome system. In contrast, unphosphorylated YAP translocates to the nucleus, modulating the transcription of genes pivotal for cell proliferation, differentiation, survival, and migration [8].

A successful pregnancy is contingent upon a cascade of sequential and discrete events, encompassing fertilization, implantation, decidualization, placentation, and parturition, each integral to the process [9]. The meticulous regulation of cell proliferation and differentiation at specific spatial and temporal junctures is indispensable. The first cell lineage specification in mammalian development occurs at the compact morula stage, culminating in a blastocyst comprising an outer trophectoderm (TE) and an inner cell mass (ICM). Subsequently, the TE undergoes gradual proliferation and differentiation to give rise to the placenta and contribute to the amnion-chorion membrane (referred to as fetal membranes) formation, a process contingent upon the equilibrium between trophoblast stem/progenitor cell proliferation and differentiation into distinct lineages. Notably, abnormalities in these trophoblast stem/progenitor cell lineages are implicated in pregnancy complications [10, 11]. Given the centrality of trophoblast cells in these processes, aberrant YAP activity has been linked to pregnancy complications like placental dysfunction and pregnancy-induced hypertension [12]. Therefore, understanding the regulatory mechanisms of YAP activity during pregnancy is paramount for elucidating the complex processes involved and developing strategies to prevent and treat pregnancy complications.

This review provides an exhaustive insight into YAP signaling, delineating its activation, regulatory mechanisms, and conjectured role in pregnancy complications. We meticulously examine the extant literature, aiming to unveil the complex interactions between YAP and pregnancy-associated pathologies, potentially illuminating pathways for innovative therapeutic interventions.

## YAP signaling pathway

**Fig. 1:**
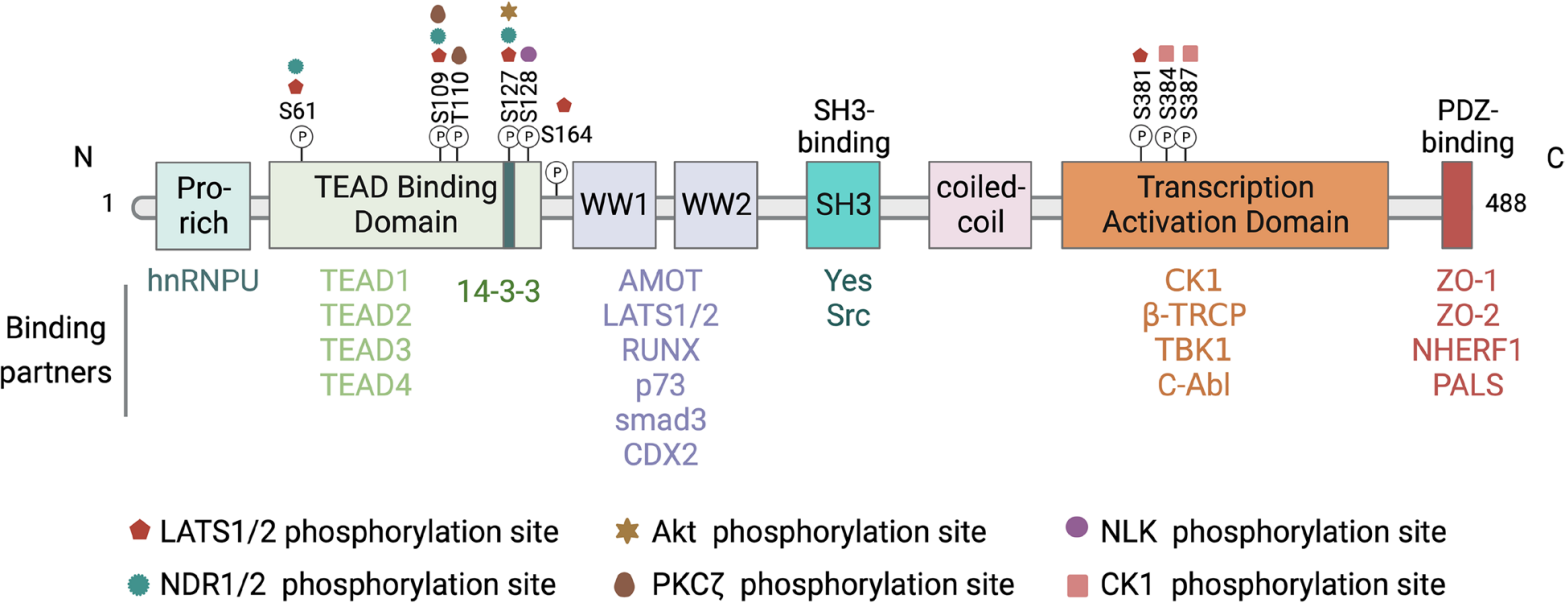
Regulatory domains of YAP. YAP protein is composed of a proline-rich domain (Pro-rich), a TEAD-binding region, a 14–3-3 binding domain, one or two WW domains (two splicing variants, YAP1 and YAP2, respectively), an Src homology domain 3- (SH3-) binding motif, a coiled-coil domain, a transcription activation domain, and a PDZ-binding motif. There are two ways in which the YAP protein is regulated, one is direct phosphorylation regulation and the other involves binding to its partner through a specific domain. S, Serine. T, Threonine

The YAP gene, located on chromosome 11q22, encodes a protein of 488 amino acids. This protein consists of various domains arranged from the NH2-terminus to the COOH-terminus, including a proline-rich domain, a TEA domain-containing sequence-specific transcription factor (TEAD-binding region), a 14–3-3 binding domain, one or two WW domains (YAP1 and YAP2 are two splicing variants, respectively), a Src homology domain 3- (SH3-) binding motif, a coiled-coil domain, a transcription activation domain (TAD), and a PDZ-binding motif [13, 14] (Fig. 1). YAP lacks a DNA binding domain, rendering it incapable of initiating gene transcription directly. It is initially known to interact with the transcription factor TEADs and serves as the primary effector of the Hippo signaling pathway [15, 16]. When the Hippo kinase module is activated, it phosphorylates the mammalian STE20-like protein kinase 1/2 (MST1/2)- Salvador homolog 1 (SAV1) complex or mitogen-activated protein kinase (MAP4Ks), resulting in the activation of the large tumor suppressor 1/2 (LATS1/2)-MOB kinase activator (MOB1A/B) complex or the nuclear Dbf2-related (NDR) kinases 1/2 (NDR1/2), respectively. Subsequently, the activated NDR1/2 and LATS1/2-MOB1A/B complex phosphorylates and inactivates YAP, leading to its retention in the cytoplasm and subsequent degradation [17–19]. Conversely, when the Hippo pathway is inactive, non-phosphorylated YAP translocates to the nucleus and stimulates gene expression by binding to the TEAD transcription factor family.

This review delves into the diverse upstream mechanisms that modulate YAP signaling activity, including the posttranslational modification of the YAP protein, the regulators that influence YAP signaling, and the interplay between YAP signaling and other signaling pathways.

### Posttranslational modifications of the YAP protein

YAP activity is intricately modulated by posttranslational modifications, which dictate its intracellular distribution and abundance (Fig. 1). A plethora of research underscores the pivotal role of serine residue phosphorylation in the switch for YAP function. LATS1/2 and NDR1/2 orchestrate the phosphorylation of YAP at specific serine residues (Ser61, Ser109, Ser127, Ser164, and Ser381 for LATS1/2; Ser61, Ser109, and Ser127 for NDR1/2) within the HxRxxS motifs [19, 20]. The phosphorylation events at Ser127 and Ser381 are crucial for modulating YAP activity. For instance, the phosphorylation at Ser127 engenders a binding site for 14–3-3 proteins, culminating in the cytoplasmic sequestration of YAP and the subsequent inhibition of its transcriptional activity [21]. In contrast, NLK-mediated phosphorylation at Ser128 fosters YAP's nuclear localization by impeding its interaction with 14–3-3 proteins [22]. Moreover, the initial phosphorylation at Ser384 and Ser387 facilitates subsequent phosphorylation by casein kinase 1 (CK1), leading to the recruitment of SCFβ-TRCP E3 ligase, YAP ubiquitination, and its eventual proteasomal degradation [23]. Interestingly, YAP activity can also be inhibited by Akt (which phosphorylates YAP at Ser127) [24] and protein kinase C ζ (PKCζ, which phosphorylates YAP at Ser109 and Thr110) [25].

Beyond the realm of phosphorylation, the PDZ-binding motif emerges as another significant player in the regulatory landscape of YAP (Fig. 1). The tight junction protein zonula occludens 2 (ZO-2) can interact with YAP's PDZ-binding motif, promoting its nuclear translocation [26]. Similarly, Na + /H + exchanger regulatory factor 1 (NHERF1) exhibits a high affinity for YAP's PDZ-binding motif [27]. Evidence suggests that the abrogation of the PDZ-binding motif attenuates YAP/TEAD-mediated transcriptional activity, underscoring its significance in YAP function [28].

### Key regulators and upstream signaling molecules of YAP

**Fig. 2:**
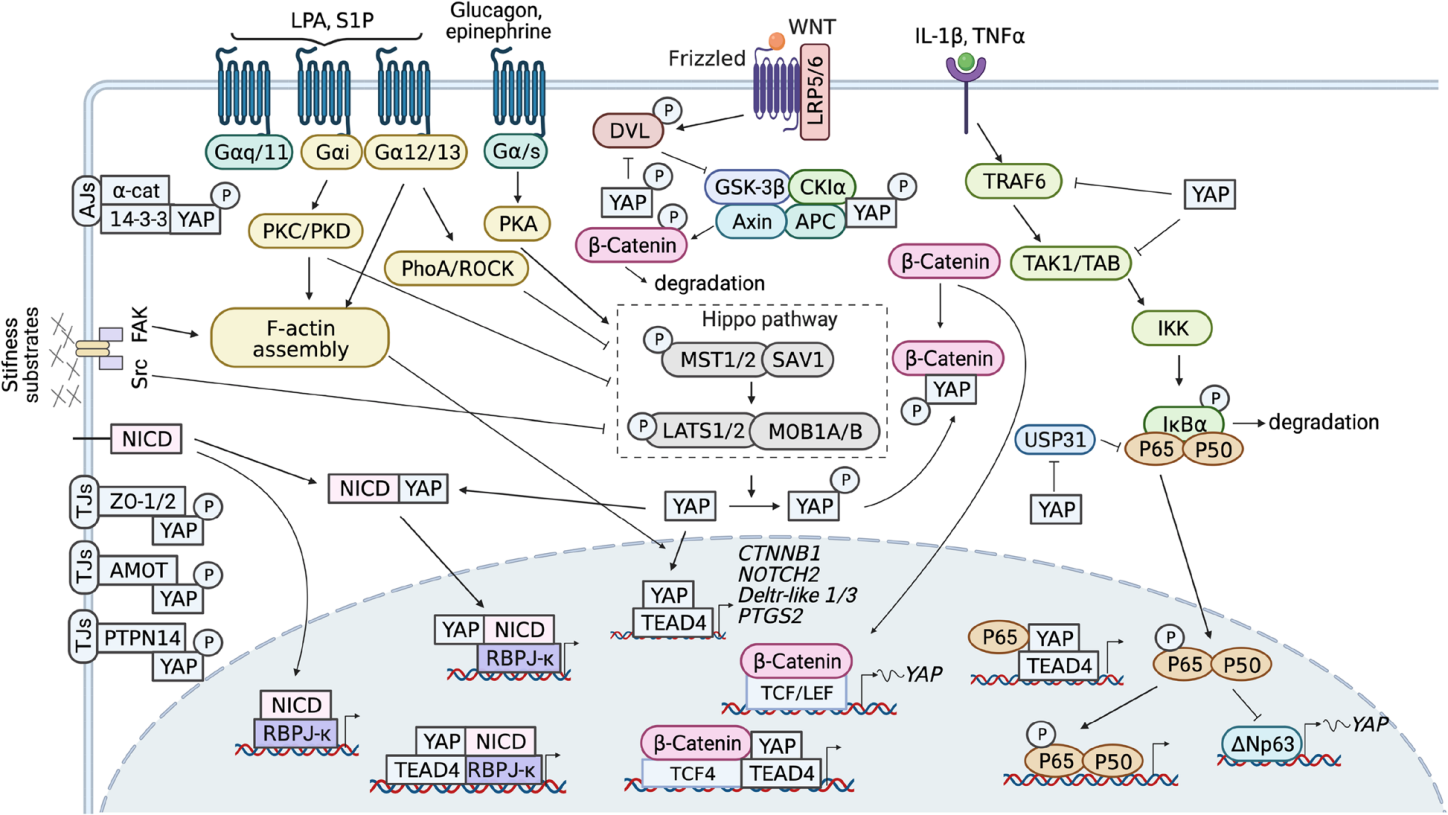
Upstream regulators of YAP. MST1/2, LATS1/2, MAP4Ks and NDR1/2 are the core components of the kinase cascade of the hippo pathway. Besides, YAP activity can be regulated by cellular context (tight junctions, adherens junctions, and soluble factors (LPA, S1P, Glucagon, and epinephrine)) and crosstalk with other signaling pathways (WNT signaling pathway, Notch signaling pathway, and NF-κB signaling pathway)

The regulation of the YAP signaling pathway is uniquely complex, given its absence of a dedicated cell surface receptor. Consequently, its modulation is intricately tied to the cellular context (Fig. 2).

#### Tight junction and adherens junction

Cellular contacts are instrumental in modulating YAP activity, a critical aspect for overseeing embryonic development and sustaining tissue architecture in mature organisms. An increase in cell density correlates with a rise in the number of tight junctions (TJs) and adherens junctions (AJs), leading to the suppression of YAP translocation from the cytosol to the nucleus. AJs, characterized by their composition of transmembrane cadherin-catenin complexes [29], facilitate the cytoplasmic retention of YAP via interactions with α-catenin [30]. In a similar vein, tight junction proteins, including the angiomotin family of proteins (AMOT), protein tyrosine phosphatase nonreceptor type 14 (PTPN14), and ZO-1/2, obstruct YAP function through their direct interactions [31–33].

#### Soluble factors

In mammals, soluble factors, encompassing hormones and growth factors, serve as conduits for transmitting organismal and distal signals from the extracellular environment, orchestrating cellular responses. G protein-coupled receptors (GPCRs), the largest family of cell surface receptors in the human genome, have the capacity to either amplify or attenuate YAP activity. This is achieved through signaling cascades initiated by heterotrimeric G-proteins, which are activated by GPCRs. Specifically, G12/13- and Gαq/11-coupled receptors, upon being stimulated by lysophosphatidic acid (LPA) and sphingosine 1-phosphate (S1P), inhibit Lats1/2 kinase activity, leading to YAP activation. In contrast, molecules like glucagon and epinephrine, signaling through Gαs-coupled receptors, enhance Lats1/2 kinase activity, resulting in the suppression of YAP function [34]. The interplay between protein kinases, Rho GTPases, and the remodeling of the actin cytoskeleton is pivotal in mediating GPCR signaling and, consequently, YAP regulation [35]. The Gα12/13-RhoA-ROCK pathway augments YAP activity by inhibiting LATS1/2-induced phosphorylation of YAP and by modulating the assembly of F-actin and myosin [36]. However, the influence of protein kinases on YAP activity is nuanced and distinct. While GPCR-PKC/PKD signaling enhances YAP nuclear localization, PKA fosters YAP phosphorylation, leading to its cytoplasmic retention [37].

### Crosstalk between YAP signaling and other pathways

Interactions between the YAP signaling pathway and other cellular signaling networks are pivotal in regulating various cellular functions. This section delineates the intricate crosstalk between YAP and other embryonic development-associated pathways, including WNT, Notch, and NF-κB signaling (Fig. 2).

#### WNT signaling pathway

The WNT signaling pathway is instrumental in orchestrating stem cell maintenance, cellular proliferation, and the determination of cell fate. In the absence of WNT ligands, β-catenin, a cytosolic protein, is targeted and phosphorylated by a heterotetrameric "destruction complex" consisting of Axin, APC, CK1, and GSK3β. This interaction facilitates the subsequent degradation of β-catenin by the β-Trcp ubiquitin ligase. However, the engagement of WNT ligands with lipoprotein receptor-related protein 5/6 (LRP-5/6) and frizzled protein (FZD) receptors on the cellular surface activates DVL, which in turn recruits the destruction complex to the receptor, elevating the cytosolic concentration of β-catenin. This accumulation of β-catenin facilitates its nuclear translocation, where it partners with T-cell-specific factor (TCF)/lymphoid enhancer-binding factor (LEF) to initiate the transcription of WNT target genes [38].

The crosstalk between YAP and WNT signaling is intricate and has been reported at various levels (Fig. 2). In the cytoplasm, YAP functions as an inhibitor of β-catenin, promoting its degradation mediated by β-Trcp through its association with the destruction complex. The presence of WNT ligands triggers the dissociation of YAP and β-catenin from the destruction complex, enabling their nuclear translocation and the subsequent activation of target genes [39, 40]. YAP also curtails enhanced WNT signaling independently of the APC/Axin/GSK3β complex, either by inhibiting DVL activity or by directly binding and sequestering β-catenin in the cytoplasm [41, 42].

In the nuclear compartment, YAP emerges as a key effector of WNT signaling. The β-catenin/TCF-4 complex augments YAP transcription by associating with its promoter [43]. There is evidence of the existence of YAP/β-catenin [44], YAP/β-catenin/TCF4 [45], and YAP/TEAD4/β-catenin [46] complexes, underscoring their substantial role in mediating cell proliferation through YAP or WNT signaling. These observations accentuate the functional overlap between YAP and β-catenin, necessitating further investigations to elucidate their shared and distinct biological roles.

#### Notch signaling pathway

The Notch signaling pathway, characterized by its evolutionary conservation, plays a pivotal role in orchestrating a myriad of developmental processes. It operates as a mechanotransduction pathway, which involves direct interactions between receptors and ligands to facilitate the transmission of cellular information between adjacent cells. The pathway comprises four distinct receptors (Notch1-4) and five ligands (Jagged 1 and 2, Delta-like 1, 3, and 4) in mammals. The activation of this pathway is initiated when a Notch receptor on one cell engages with a Notch ligand on an adjacent cell [47]. This interaction triggers the sequential cleavage of the Notch receptor by ADAM-family metalloproteases and γ-secretase, releasing the Notch intracellular domain (NICD). The NICD then migrates to the nucleus, where it associates with recombining binding protein suppressor of hairless (RBP-Jκ, also known as CSL or CBF1), instigating the transcription of Notch target genes, including those encoding basic helix-loop-helix (bHLH) transcriptional repressors like Hes/Hey [48].

The crosstalk between YAP and Notch signaling has been substantiated by numerous studies, underscoring their intertwined regulatory mechanisms (Fig. 2). YAP augments the Notch signaling pathway by enhancing the expression of Notch ligands and receptors, such as Notch1/2/3, Jagged 1/2, and Delta-like ligands 1/3 [49, 50]. However, the intricate molecular mechanisms underpinning this regulation are yet to be fully elucidated. A handful of studies have documented YAP’s capability to associate with the promoter of Notch2 and the distant enhancer of Delta-like ligands [49, 51]. Furthermore, YAP has been observed to interact with NICD physically, forming the YAP-NICD complex, which is recruited to chromatin by RBP-Jκ, contributing to the modulation of Notch signaling [52].

Reciprocally, Notch signaling exerts influence over YAP signaling [53]. A comprehensive mice genome-wide study utilizing ChIP-Seq and transcriptome analyses pinpointed YAP as a direct target of the RBPJ/N1ICD complex. Remarkably, the expression of YAP is potent enough to counteract the inhibition of the Notch pathway in neural stem cell self-renewal assays [54]. A recent study unveiled an activated Notch-YAP circuit that fosters stemness and tumorigenesis in embryonal rhabdomyosarcoma. In this context, Notch signaling elevates both YAP gene expression and activity, while YAP reciprocally boosts the transcription of JAG1, DLL1, and RBPJ mRNA levels [55]. These findings highlight the functional redundancy between YAP signaling and Notch signaling. Elucidating the primary and secondary relationships of these signaling pathways and the molecular mechanisms underlying their crosstalk under distinct physiological conditions holds a significant interest.

#### NF-κB signaling pathway

The NF-κB signaling pathway plays a crucial role in mediating inflammatory responses. In its inactive state, NF-κB is sequestered in the cytoplasm by inhibitor kappa B (IκB) proteins. Various stimuli, including proinflammatory cytokines like IL-1β and TNF-α, activate transforming growth factor-beta-activated kinase 1 (TAK1) and the IκB kinase complex, leading to IκBα phosphorylation, ubiquitination, and degradation. This process liberates NF-κB, allowing its translocation to the nucleus to regulate a plethora of target genes. The classic NF-κΒ family consists of NF-κB1 (p105/p50), NF-κB2 (p100/p52), p65 (RelA), c-rel, and RelB. Homodimers or heterodimers of these family members assemble into active NF-κΒ transcription factors [56].

The interaction between YAP and NF-κB signaling is multifaceted. Proinflammatory cytokines TNF-α and IL-1β have been shown to inhibit YAP expression in a concentration-dependent manner, a process potentially mediated by p65/NF-κB inhibiting ΔNp63 [57]. On the flip side, increased YAP levels can counteract inflammation by inhibiting NF-κB signaling [58]. YAP achieves this by enhancing the transcription of IκBa [59] and interacting with key upstream components of the NF-κB pathway, including TAK1 and TRAF6 [60, 61]. Moreover, YAP can suppress the transcription of NF-κB target genes like cyclooxygenase 2 (COX2), especially at low cell densities, by recruiting HDAC7 to the COX2 promoter region in conjunction with TEAD, even in the presence of IL-1β and TNF-α-induced NF-κB activity [62] (Fig. 2).

Interestingly, a positive regulatory relationship also exists between YAP and NF-κB signaling (Fig. 2). In adult T-cell leukemia/lymphoma cells, Tax-induced p65 activation interrupts the YAP-LATS1 interaction, preventing YAP phosphorylation. Activated p65 then associates with YAP to enhance the expression of YAP target genes [63]. YAP and p65 interaction also plays a pivotal role in modulating the macrophage inflammatory response to lipopolysaccharide (LPS) stimulation [64]. IKKβ/ε has emerged as a novel modulator of YAP phosphorylation, with activated YAP and NF-κB working in tandem to regulate the transcription of downstream genes [65]. Furthermore, YAP augments NF-κB signaling by inhibiting the expression of ubiquitin-specific peptidase 31 (USP31), a potent NF-κB inhibitor [66].

## The role of YAP in trophoblast cells

The TE constitutes the outer layer of the human blastocyst, giving rise to both the placental and fetal membrane trophoblast post-implantation. However, due to ethical constraints and limited human models, our understanding of the TE's early developmental stages is limited. Early TE growth and differentiation have been delineated through descriptive studies of human embryonic material [67, 68], bulk transcriptome analyses, and in vitro research employing primary cells, cell lines, and villous explants.

Approximately 4–5 days post-fertilization, the TE emerges, marking the inaugural cell fate specification and distinguishing the TE from the ICM [67]. The TE is characterized by its polarity. The polar trophectoderm, a distinct segment of the trophectoderm adjacent to the ICM, binds to the receptive endometrial epithelium around 5–6 days post-fertilization, initiating human placental development. Notably, polar trophectoderm cells, at the onset of implantation, exhibit invasive and proliferative traits, unlike their distal trophoblast counterparts [69].

Upon establishing a stable connection with the maternal endometrium, the polar trophectoderm differentiates into the first trophoblast lineages: the multinucleated primitive syncytium (PS) [70]. Concurrently, the cytotrophoblast cells proliferate swiftly, forming projections that pierce the PS, leading to the creation of primary villi that delve into the maternal decidua, eroding its blood vessels and glands. The epithelial surface undergoes branching and expansion due to the continuous proliferation and fusion of emerging villous cytotrophoblast (CTB). This results in the formation of the outer syncytiotrophoblast (STB) layer, which interfaces directly with maternal blood, facilitating the exchange of oxygen, nutrients, and waste.

In addition to chorionic villi development, CTBs at distal locations spread laterally, constructing the trophoblastic shell. This structure serves as the origin for the second differentiated trophoblast cell type, the extravillous trophoblasts (EVT). Once mature villi are established, EVTs derive from the differentiation of CTBs at the tips of anchoring villi. In the cytotrophoblastic cell column, proximal cell column trophoblasts exhibit a proliferative phenotype, representing EVT lineage progenitors [71]. In contrast, distal cell column trophoblasts differentiate into EVTs, which widely lose their replicative capability, transitioning into a senescent state [72]. Functionally, EVTs can be further categorized into interstitial EVTs, which anchor the placenta by invading the uterine wall, and endovascular EVTs, which permeate maternal decidual arterioles and glands, enhancing nutrient and oxygen transport [73]. Trophoblast lineage differentiation persists throughout gestation and remains consistent until term. Any anomalies in trophoblastic differentiation can lead to pregnancy complications, such as miscarriages [74].

Conversely, insights into the trophoblast lineage in the smooth chorion are sparse. Smooth chorionic trophoblast cells are organized in an epithelial configuration. Prior research indicates that smooth chorionic CTBs penetrate the uterine wall, promoting the fusion of the smooth chorion with the parietal decidua [75]. Unlike placental EVTs, which remodel maternal arteries, smooth chorionic CTBs refrain from invading maternal blood vessels in the decidual tissue [75]. Recent single-cell RNA sequencing of the smooth chorion has identified CTBs, EVTs, and STBs subtypes. Notably, CTB 4 emerges as a unique subtype exclusive to the smooth chorion, distinct from the villous chorion [76], implying divergent functions between the placental chorion and the smooth chorion.

Recently, the advent of the human trophoblast stem cell model [77], trophoblast organoids [78], and stem cell embryo models, including blastoids [79], has facilitated investigations into the role of transcription factors in human trophoblast differentiation. In the human blastoids model, atypical protein kinase C (aPKC) and F-actin expression domains were observed to align in outer cells, which also exhibited nuclear YAP accumulation. TE specification and morphogenesis are contingent upon aPKC, Hippo pathway inhibition, YAP's nuclear translocation, and its binding affinity to TEAD transcription factors [79]. Furthermore, various studies have underscored the implications of aberrant YAP activity on trophoblast lineage development and cellular dysfunction.

This review delves into YAP's influence during trophoblast lineage evolution.

### YAP in trophoblast differentiation

**Fig. 3:**
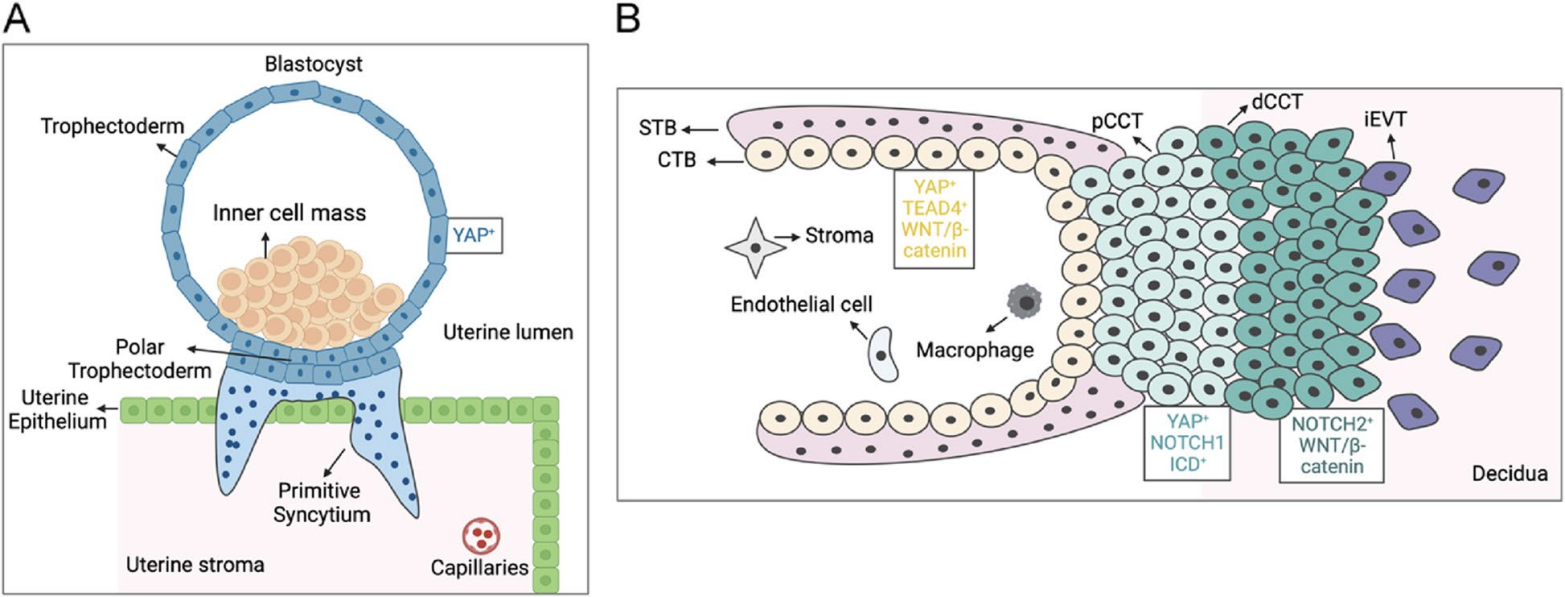
The dynamic changes of YAP activity in trophoblasts during the formation of human placental anchoring villus. **A** At the blastocyst stage, the trophectoderm has high YAP activity. At the beginning of embryo implantation, trophoblast located in the polar trophectoderm comes into contact with uterine epithelial cells and rapidly proliferates and fuses to form the invasive primitive syncytium, which is the first event in trophoblast specification. **B** The dynamic changes of YAP, NOTCH, and WNT/β-catenin signaling in the development of placental anchoring villus. CTB, cytotrophoblast. STB, syncytiotrophoblasts. pCCT, proximal cell column trophoblast. dCCT, distal cell column trophoblasts. iEVT, interstitial extravillous trophoblast

The initiation of the pre-implantation trophectoderm (TE) program occurs in the outer cells of the morula stage. These cells establish apical-basal cell polarity, a process governed by the activation of aPKC, which in turn modulates the expression and nuclear translocation of YAP and GATA-binding protein 3 (GATA3). This mechanism is conserved across human, bovine, and murine species [80]. By the blastocyst stage, the YAP/TEAD complex, localized in the nuclei of outer cells, induces the expression of caudal-type homeobox transcription factor 2 (CDX2), facilitating human TE specification. This observation aligns with previous findings in mouse blastocysts. In mouse ICM, YAP is phosphorylated and confined to the cytoplasm, a process yet to be thoroughly examined in humans [79, 81] (Fig. 3).

In mouse embryos, the YAP/TEAD4 complex is known to modulate the transcriptional activity of CDX2, in conjunction with the Notch signaling pathway [82]. Strawberry Notch1 (Sbno1), a highly conserved chromatin factor, has been identified in mice to influence the trophectoderm-enhancer (TEE) of CDX2, ensuring its robust activation by the YAP-TEAD4 and NICD-RBPJ complexes [83]. This differential YAP signaling is pivotal in determining the fate specification of trophoblast cells during TE formation.

The TE serves as a precursor to both placental villous trophoblast and the smooth chorionic trophoblast cells. Aberrations in trophoblast differentiation can precipitate placentation failures and subsequent fetal and maternal complications [84]. Differentiated human trophoblasts, encompassing chorion trophoblast cells (CTCs), CTBs, STBs, and EVTs, execute diverse functions throughout pregnancy. Evidence suggests that CTB growth and/or cell fusion are compromised in cultures derived from human placentas associated with preeclampsia (PE) or fetal growth restriction (FGR) [85, 86]. Moreover, CTBs sourced from preeclamptic placentas display defects in EVT formation in vitro [87].

The regulatory elements steering human placental differentiation remain elusive. Emerging research posits YAP as a potential regulator of trophoblast lineage formation and placental expansion. YAP exhibits varied expression across different trophoblast populations and interacts with a unique set of transcription factors in the early human placenta [88]. It is notably absent in hormone-producing STBs but is strongly expressed in CTBs and cell column trophoblasts (CCTs) of early human placenta, with muted expression in EVTs [89]. The YAP/TEAD complex is instrumental in preserving CTB stemness and also modulates the differentiation of CTBs into STBs and EVTs. Recent insights from studies utilizing primary cells, three-dimensional organoids, and CRISPR-Cas9 genome-edited JEG-3 clones have unveiled that human CTBs can spontaneously differentiate into syncytiotrophoblast-like cells. The inhibition of YAP/TEAD activity is integral to this differentiation process [89]. Further exploration of the early human placenta has highlighted the role of S100P and cAMP signaling-induced YAP activity inhibition in triggering the syncytialization of CTB-derived human TSCs [90, 91].

The modulation of WNT downstream effectors and NOTCH receptor expression in human first-trimester placental tissues is instrumental in directing the differentiation of EVT progenitors into EVTs [71, 92]. NOTCH signaling components, akin to the YAP expression pattern, display varied expression across distinct trophoblast subtypes within the placental villus. NOTCH1 ICD is identifiable in EVT progenitors, while NOTCH2 is predominantly expressed in EVTs [71, 93]. YAP, potentially through its interaction with Notch1 or Notch2, is implicated in the regulation of CTB differentiation into EVTs and EVT progenitor cell differentiation. The activation of canonical WNT signaling plays a crucial role in TSC and/or CTB progenitor expansion, as well as the regulation of EVT migration and differentiation [94]. It is speculated that the crosstalk between YAP transcription factors and WNT signaling is deemed essential for balancing TSC and/or CTB progenitor expansion and EVT differentiation. In murine TSCs derived from blastocysts, nuclear YAP accumulation is observed, while it is primarily localized in the cytoplasm in differentiated trophoblast cells [95].

### YAP-mediated stemness maintenance and proliferation of trophoblast cells

YAP contributes to sustaining the stemness and proliferation of CTB progenitors, mediated through intricate genomic mechanisms. In the developing human placenta, YAP-TEAD4 complexes are pivotal in activating genes associated with the cell cycle and stemness. Concurrently, they repress genes implicated in trophoblast cell fusion, thus promoting CTBs growth and expansion [89]. The role of the YAP-TEAD4 complex can extend to being a vital regulator of murine TE development, where it activates CDX2 and other key TE regulators in the outer cells of preimplantation embryos [81]. In murine TSCs, the nuclear translocation of YAP facilitates its WW2 domain to interact with the PPQY motif of CDX2. This interaction is central to modulating trophoblast proliferation by downregulating CyclinD1 levels [95]. These findings indicate that the nuclear presence of YAP not only augments trophoblast cell expansion but also intrinsically moderates trophoblast proliferation. The equilibrium between pro-proliferative and inhibitory actions mediated by YAP is fundamental to trophoblast lineage differentiation and fostering a healthy pregnancy. Therefore, positioning YAP as a prospective target for interventions in hyperplastic trophoblast disorders, albeit necessitating further research.

The activation of the WNT signaling pathway may be necessary for in vitro TSC/CTB progenitor expansion. Yet, the principal components of this pathway in vivo are to be fully delineated. Nuclear recruitment of YAP in the Hippo-off state could destabilize the cytoplasmic β-catenin destruction complex, culminating in β-catenin's nuclear accumulation. YAP may also interact with TCF-1 to ensure the self-renewal of CTBs in cytotrophoblast organoids from the human placenta [96]. Moreover, Wnt3a has been implicated in maintaining bovine TSCs, modulating CDX2 expression via the WNT-YAP signaling pathway [97]. In essence, the interplay between WNT signaling and YAP is anticipated to be vital for TSC expandability. YAP underscores its significance in maintaining cell proliferation and stemness, warranting extensive studies to comprehensively unravel its intricate mechanisms and broader implications.

### YAP in trophoblast invasion

Abnormal placental development has profound implications for both maternal and fetal health, with inadequate trophoblast invasion often linked to severe conditions such as PE and FGR [87, 98]. A notable reduction in YAP expression in preeclamptic placentas underscores the integral role of YAP signaling in the etiology of PE, particularly in modulating trophoblast invasion [99, 100].

Empirical studies elucidate that YAP overexpression enhances cell invasion capabilities in BeWo, HTR-8/SVneo which is a heterogeneous population consisted by trophoblast and stromal/mesenchymal cells, and JAR cells [100]. In a nuanced interaction, miR-326 suppresses trophoblast cell (HTR-8/SVneo and JEG-3 cells) growth, invasion, and migration by targeting PAX8-mediated YAP expression [101]. The activation of the Hippo/YAP signaling pathway in human trophoblasts from PE-complicated pregnancies is linked to inhibiting trophoblast invasion and migration. This inhibition is mediated by the upregulation of miR21, which impedes PP2A β function, leading to LATS1-YAP phosphorylation and the subsequent cytoplasmic retention of YAP, thereby restraining EVT invasion and migration [102].

It has been evident in the crosstalk between Notch and WNT signaling in cell migration and invasion. Notch2 signaling, for instance, has been implicated in attenuating trophoblast migration in human distal cell column trophoblasts. Conversely, Wnt3A enhances the migration and invasion of trophoblast cells isolated from the early placenta by magnetic bead sorting, an effect that can be mitigated by Dickkopf-1 [93, 103]. The complex interactions among Notch, WNT, and YAP signaling pathways are anticipated to be central in trophoblast invasion, necessitating comprehensive research to unravel their synergistic and antagonistic mechanisms.

## YAP and pregnancy complications

The intricate balance of trophoblast cell proliferation and differentiation is fundamental to the development of the placenta [104]. The human placental STB are instrumental in fostering maternal immune tolerance through secreting immunosuppressive proteins such as PD-L1 and type III IFNs and exhibit an enhanced resistance to infection, being 20-fold more resilient than CTBs [105–107]. In parallel, research involving rodents and HTR-8/SVneo cells indicates that EVTs possess a unique ability to induce a regulatory phenotype in decidual immune cells, promoting fetal tolerance [108].

Beyond its immune functions, the chorion, as a barrier, is integral in modulating intrauterine prostaglandin (PG) concentrations and metabolism. This is particularly evident in the role of human amniotic membrane-derived PGs in initiating labor through the facilitation of cervical ripening and myometrial contractions [109, 110]. NAD-dependent 15-hydroxy-PG dehydrogenase (15-PGDH), an enzyme responsible for converting active PGs into inactive forms, is predominantly expressed in the chorionic trophoblast layer. Notably, lower expression levels of 15-PGDH are observed in spontaneous labor at term compared to elective caesarean sections, with a further reduction noted in preterm labor without infection [111]. Moreover, the intensity and number of 15-PGDH-positive cells are markedly reduced in the chorionic trophoblast layer of preterm patients with diagnosed infection compared to idiopathic preterm patients without a diagnosed infection [112].

Mounting evidence suggests that dysregulation of biochemical, endocrine, and immunological processes at the maternal–fetal interface, particularly chorionic dysfunction, is linked to adverse pregnancy outcomes. The restricted proliferation of human trophoblast stem cells (TSCs) in early pregnancy correlates with recurrent spontaneous abortion (RSA) [113]. Concurrently, anomalies in the proliferation, migration, and invasion of trophoblast cells are implicated in severe pregnancy complications, including FGR and PE [114, 115]. Notably, abnormal trophoblast differentiation is also a key factor in pregnancy complications, as CTBs extracted from the placentae of preeclamptic patients have been observed to exhibit defects in EVTs formation [87], and impairments in CTBs growth and cell fusion are noted in cases of PE and FGR [89].

To enhance the understanding of YAP's role in these complications, future research should focus on elucidating the specific mechanisms through which YAP signaling influences trophoblast cell functions, immune interactions at the maternal–fetal interface, and the onset of labor, providing insights for potential therapeutic interventions.

### Spontaneous miscarriage

Miscarriage, defined as the spontaneous loss of a pregnancy before 24 weeks of gestation, is a common complication, affecting approximately 15% of all pregnancies [9]. During the first trimester, a significant proportion of early miscarriages are attributed to impaired placentation due to developmental or functional anomalies in the trophoblastic lineage and chromosomal abnormalities [116, 117]. A subset of placentae from patients with idiopathic recurrent pregnancy loss (RPL) displays compromised CTBs/STBs bilayer formation and defective trophoblastic column formation [118].

A marked reduction in YAP activity has been observed in the villi tissue of miscarriage samples. In healthy pregnancies (HP) around 6–8 weeks of gestation, YAP and Ki-67 are primarily localized in the nucleus of CTBs within the villous tissue. In contrast, in RSA cases of similar gestational age, phosphorylated YAP (p-YAP) is predominantly found in the cytoplasm of CTBs. This elevation in p-YAP levels is facilitated by the suppression of CDC42/EZRIN signaling, leading to the human TSCs differentiation and the inhibition of their proliferation [113]. Additionally, evidence suggests that the absence of TEAD4 curtails the self-renewal of both mouse and human CTB progenitors by downregulating essential cell cycle gene expression [118], underscoring YAP's role in modulating CTB proliferation through the activation of TEAD4-mediated gene expression.

Increased YAP activity in CTBs is also associated with RPL. Patients experiencing RPL have been found to have reduced serum S100P levels compared to healthy individuals. The inadequate levels of S100P are unable to inhibit YAP/TEAD signaling effectively, resulting in the sustained progenitor status of CTBs and hampering the trophoblast syncytialization of CTBs-derived human TSCs [91]. However, the specific mechanism through which S100P inhibits YAP/TEAD signaling in the human placenta remains to be elucidated.

Excessive inflammation is recognized as a contributing factor to miscarriage [119]. Elevated levels of TNF-α have been detected in the serum of women undergoing miscarriage [120]. Rodent studies have shown that mouse blastocysts, when pre-treated with TNF-α in vitro, exhibit increased mortality rates upon transfer into pseudopregnant mice. Additionally, the nucleoplasmic ratio of NF-κB p65 in villous stromal cells of the human placenta from early spontaneous abortions is significantly elevated compared to controls [121]. It has been reported that NF-κB activation augments CXCL8 expression and triggers the release of TNF-α and IL-1β, instigating unexplained RPL through the activation of the inflammatory response in human placental trophoblasts [122]. Moreover, a result of immunological staining has reported higher levels of tumor necrosis factor receptor 1 (TNFR1) in villous stromal cells from early spontaneous abortion samples compared to those from normal pregnancies of matched gestational age, while TNFR1 levels in placental trophoblasts did not exhibit significant variations between the two groups [123]. These insights underscore the role of the activated TNF-α/TNFR1/NF-κB signaling pathway in villous stromal cells and placental trophoblasts in precipitating immunological pregnancy loss.

### Preeclampsia

Preeclampsia is a complex disorder diagnosed by the onset of hypertension (BP > 140/90 mmHg) and proteinuria (> 300 mg/24 h) post the 20 weeks of gestation. It is often accompanied by renal and liver dysfunction, uteroplacental insufficiency, and FGR [115]. The condition is partly attributed to impaired trophoblast invasion, along with insufficient spiral arterial remodeling, oxidative stress, and trophoblast dysfunction [124, 125].

YAP has been implicated in PE development. A significant reduction in both mRNA and protein levels of YAP has been observed in placentas from patients with severe PE (sPE) compared to those from normal pregnancies [100]. Further investigation revealed a notable decrease in the number of CTBs and EVTs with high YAP nuclear expression in PE placentas compared to age-matched controls, suggesting inactive YAP signaling at the onset of PE [102, 114]. The elevated expression levels of MST1/2 in sPE placentas further suggest that YAP signaling inactivation occurs through the dual mechanisms of mRNA level reduction and YAP protein degradation induced by Hippo signaling [100].

YAP dysfunction is increasingly recognized for its role in PE development, particularly by inhibiting cytotrophoblast invasion. In HTR-8/SVneo cells, 17β-estradiol (E2) stimulates G protein-coupled estrogen receptor (GPER) activation, promoting YAP nuclear translocation and subsequently enhancing trophoblast cell invasion via the upregulation of angiopoietin-like 4 (ANGPTL4) [114]. Given the reported lower serum E2 levels in PE patients, a deficiency in E2 synthesis or signaling is suggested as a contributing factor to PE development [126]. Consequently, downregulation of the GPER/YAP/ANGPTL4 axis due to decreased estrogen levels exacerbates trophoblast cell invasion impairment, intensifying PE pathogenesis [114].

Sphingosine-1-phosphate (S1P), another GPCR ligand, is implicated in regulating YAP activity in trophoblasts affected by PE. While S1P synthesis and expression are typically abundant in normal trophoblasts, the placentas of PE mice exhibit reduced S1P levels. Sphingosine-1-phosphate receptor-2 (S1PR2), a GPCR located on the membrane, acts as a known receptor of S1P [127]. S1P enhances HTR8/SVneo cell invasion in a YAP-dependent manner, stimulated by S1PR2 and downstream Rho/ROCK-induced actin polymerization. However, diminished S1P expression in PE mice placentas attenuates this YAP activity, resulting in compromised EVTs invasion [12].

The role of microRNAs (miRNAs) in modulating YAP activity is also evident. Elevated levels of microRNA let-7a in human early-onset sPE placentas inhibit YAP levels by directly binding to its 3' UTR, leading to increased apoptosis in JEG-3 cells [115]. Concurrently, enhanced miR21 levels in EVTs from human PE pregnancies are associated with reduced trophoblast invasion. In vitro, miR21 suppresses HTR-8/SVneo cell invasion and migration by elevating cytoplasmic p-YAPSer127 and LATS1Thr1079 levels, mediated by the reduction of PP2A β levels [102].

During PE, the placenta is exposed to excessive oxidative stress and inflammation due to insufficient spiral arterial remodelling, leading to increased NF-κB activity [128]. NF-κB protein levels in the placenta of women with PE were significantly higher than those in normotensive pregnant women [129]. In first-trimester human villous explants, LPS treatment upregulates IL-6, IL-1β, IL-8, RANTES, and TNF-α levels, inducing trophoblast cell apoptosis [130]. Targeting NF-κB can ameliorate LPS-induced trophoblast dysfunction [131], and antagonizing the TLR4 signaling pathway can improve the LPS-induced PE-like phenotype in rodents associated with NF-κB inactivation in the placenta [132]. Thus, inhibiting the TLR4/NF-κB signaling pathway emerges as a promising therapeutic strategy for PE, mitigating inflammation-induced pyroptosis [133].

### Preterm birth

Preterm birth, characterized by deliveries occurring between 28 and 37 weeks of gestation, is a complex condition with multifactorial etiologies. The involvement of YAP, though not directly established, can be inferred through its interaction with molecules and pathways implicated in preterm birth.

NF-κB, a pivotal player in inflammatory responses, has been studied in the context of fetal membranes. Research utilizing an ex vivo model of perfused full-thickness term fetal membranes revealed augmented nuclear translocation of p65 in human CTCs following LPS treatment on the decidual surface [134]. NHERF1, a negative regulator of YAP activity, is implicated in escalating the release of proinflammatory cytokines mediated by NF-κB across various cell types [135, 136]. In a comparative study between preterm and term births focusing on the human fetal membrane's amnion and chorion layers, elevated NHERF1 protein levels were discerned in the preterm group. This observation is corroborated by in vitro findings where LPS treatment escalated NHERF1 levels in human primary CTCs. An inflammation-associated preterm labor mouse model, subjected to LPS treatment, exhibited preterm delivery within 24 h and heightened NHERF1 levels in fetal membranes, underscoring the potential link between YAP inactivity, NHERF1 elevation, and NF-κB signaling activation in inflammation-induced preterm birth [137]. However, this hypothesis requires further support from direct data.

Tight junctions and adherens junctions play crucial roles in maintaining the physical barrier function of epithelial cells. YAP participates in tight junction formation by interacting with the tight junction-related protein ZO-1, which subsequently regulates cell migration [138]. ZO-1 deficiency in mouse embryos precipitates a lethal phenotype marked by impaired yolk sac angiogenesis and increased embryonic cell apoptosis [139]. During pregnancy, ZO-1 participates in human trophoblast differentiation and placental defense mechanisms [140, 141]. Predominantly found in human placental CTBs, reduced ZO-1 expression is associated with the transition of CTBs to STBs, mirroring YAP's inhibitory effect on trophoblastic syncytialization [89, 141]. In normal pregnancies, ZO-1 expression is evident in both human amniotic epithelial cells and chorionic trophoblast cells. However, a negative correlation emerges between the severity of intrauterine infection and ZO-1 expression in chorionic trophoblast cells, not in the amniotic epithelium [142]. The intricate dance between YAP, NF-κB, NHERF1, and ZO-1 in the context of preterm birth underscores the necessity for comprehensive investigations.

### Fetal growth restriction

Fetal growth restriction, also known as intrauterine growth restriction (IUGR), refers to the failure of a fetus to achieve its genetic growth potential. It is a common pregnancy complication associated with multiple adverse perinatal outcomes. While various factors contribute to the pathophysiology of FGR, impaired placentation is considered the primary and most prevalent cause due to the crucial role of the placenta in providing optimal conditions for fetal growth in utero [143]. Deficiencies in extravillous trophoblast invasion and maternal arterial remodeling are central to placental dysfunction in FGR [144].

YAP expression dynamics are notable in the context of FGR. While YAP is expressed in CTBs, it is markedly downregulated or degraded in STBs. The human FGR placentas exhibit increased YAP phosphorylation compared to their normal counterparts. A study utilizing an ERK inhibitor-induced FGR mouse model revealed elevated placental p-YAP levels and diminished expression of YAP target genes, including CTGF, CYR61, and AMOTL2. In vitro experiments underscored the potential of upregulated YAP to mitigate the ERK inhibition-induced impairment of HTR-8/SVneo cells invasion and migration [145]. The role of maternal vitamin D deficiency (VDD) in IUGR is highlighted by its association with enhanced YAP phosphorylation. Mice subjected to a VDD diet exhibited a series of placental abnormalities, including a thinner labyrinth, trophoblast necrosis, and cytotrophoblast vacuolar degeneration. These pathological changes were concomitant with increased YAP phosphorylation. In vitro studies further elucidated VDD's role in suppressing human trophoblast cell invasion and promoting EVT apoptosis, mediated by the activation of the Hippo-YAP signaling pathway [146]. These findings underscore the intricate relationship between YAP signaling and FGR.

## Conclusion and perspectives

**Fig. 4:**
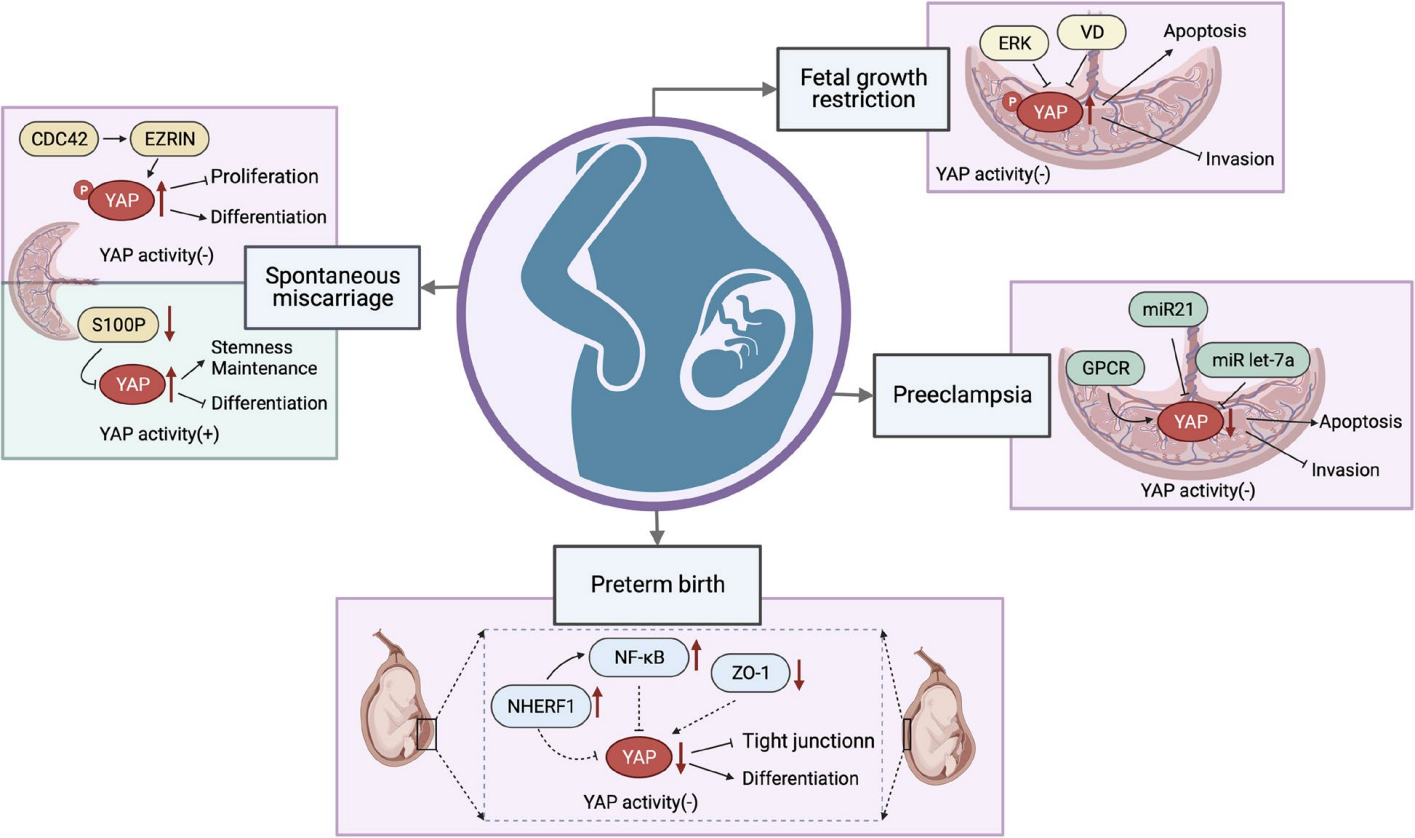
Aberrant YAP function in trophoblasts is involved in the pathological mechanism of pregnancy complications. The schematic diagram shows the association of abnormal YAP activity in trophoblasts with pregnancy complications such as preeclampsia, fetal growth restriction, miscarriage, and preterm birth. Lines indicate proven. Dotted lines indicate presumed

The integrity of the maternal–fetal interface is pivotal for a healthy pregnancy, yet its disruption is associated with a range of pregnancy complications, including miscarriage [147], PE [148], and PTB [148]. This disruption is often linked to placental and fetal membrane dysfunction, characterized by the abnormal proliferation and differentiation of trophoblast cells [149]. In this review, we highlighted the intricate role of Yes-associated protein (YAP) in this context, illuminating its influence on trophoblast cell dynamics and subsequent pregnancy outcomes (Fig. 4). In studies on placental tissues from abortion cases, we encountered conflicting data on YAP expression in CTBs. While one study associated reduced nuclear YAP levels with inhibited CTB proliferation leading to abortion, another attributed increased nuclear YAP levels to disease occurrence by impeding cell differentiation. These contrasting findings underscore the complex and multifaceted role of YAP in the placenta of pregnancy complications. Based on the dynamic shifts in YAP signaling during CTB to EVT differentiation, we hypothesize that the deregulation of YAP activity, which inhibits EVT differentiation, maybe a secondary effect stemming from its influence on CTB and trophoblast stem cell proliferation. Given that YAP affects the proliferation of CTB and trophoblast stem cells, and deregulation of the proliferation rate contributes to the dysfunction of the progenitor pool and the development of pregnancy complications. However, ethical constraints and the lack of comprehensive human models render the progenitor pool during this differentiation a "black box," leaving the mechanisms by which YAP disrupts this pool largely unexplored.

Inflammation plays a dual role in pregnancy, being both a necessity for embryo implantation and pregnancy maintenance and a potential threat when dysregulated [150]. Our review identified activated NF-κB signaling in villous CTBs associated with spontaneous miscarriage and PE. The intricate interplay between YAP and inflammation is underscored by their mutual regulatory dynamics and the diverse cellular responses elicited. Evidence from various studies underscores YAP's protective role in cellular and tissue contexts under inflammatory conditions. A case in point is a mouse model of bacterial pneumonia, where YAP facilitated the proliferation and differentiation of alveolar epithelial cells’ stem/progenitor cells post-infection, aiding in tissue repair and regeneration [59]. Similarly, in hepatocytes, YAP's role is concentration-dependent; low TNF-α levels promote YAP nuclear translocation and cell proliferation, whereas higher concentrations induce YAP phosphorylation and inactivation, culminating in apoptosis [151]. While these insights are not directly derived from studies on placental or fetal membrane trophoblast cells, the structural similarities these cells share with the studied tissues warrant the extrapolation of these findings. The protective role of YAP against inflammation, particularly in placental trophoblast cells, emerges as a critical area for further investigation. In the context of LPS-induced vascular injury, the dual role of YAP is again evident. While LPS induces endothelial cell pyroptosis, it also inhibits the proliferation of surviving cells by promoting YAP phosphorylation and inactivation, a process contributing to inflammatory lung injury [152]. These findings suggest that even under inflammatory conditions, some cells remain viable but exhibit altered activity compared to normal cells, which can manifest as impaired YAP-induced cell proliferation-mediated repair capacity. The ambiguity extends to the realm of pregnancy, where the impact of inflammation on YAP-regulated functions in surviving trophoblast cells is yet to be fully elucidated. Early pregnancy is characterized by a delicate balance, where a moderated inflammatory environment is conducive to trophoblast lineage differentiation and the establishment of the maternal–fetal interface. However, an excessive inflammatory response precipitates trophoblast cell pyroptosis [133, 150]. The nuanced modulation of YAP activity in response to varying inflammatory concentrations emerges as a pivotal aspect warranting comprehensive investigation. Unraveling this complexity could illuminate targeted interventions to mitigate the adverse impacts of inflammation on pregnancy outcomes.

Excitingly, several drugs in clinical use can already restrict YAP activity, and several novel YAP inhibitors are under development. Broadly categorized based on their target pathways, YAP inhibitors can be classified into three groups: those targeting upstream regulators of YAP activity, those targeting YAP/TAZ or TEADs and disrupting their interaction, and those targeting downstream YAP transcriptional target genes with oncogenic effects [153]. Several YAP inhibitors have progressed to the first phase of clinical trials, marking a significant milestone in this field. These include the antisense oligonucleotide inhibitor ION537 (NCT04659096) by Ionis Pharmaceuticals, VT3989 by Vivace (NCT04665206) for solid tumors, and Novartis' IAG933 (NCT04857372), a proprietary compound delineated in patent WO2021186324A1, for neuromas. Among the FDA-approved drugs, statins have emerged as potent YAP inhibitors [154]. These 3-hydroxy-3-methylglutaryl coenzyme A (HMG-CoA) reductase inhibitors are known to suppress YAP activity by inhibiting its nuclear translocation and augmenting cellular sensitivity to other inhibitors, especially in the context of solid tumors [155]. In the realm of pregnancy complications like PE, statins have been employed to manage hypercholesterolemia, with recent studies attesting to their safety and dispelling concerns over potential teratogenic effects [156–158]. However, a meta-analysis encompassing nine studies revealed an uptick in spontaneous abortion rates among women subjected to statin therapy during pregnancy [159]. Another study correlated statin exposure to increased incidences of preterm labor and low birth weight [160]. These adverse outcomes are hypothesized to be tethered to the attenuation of YAP activity at the maternal–fetal interface, accentuating the vulnerability of trophoblast cells. In this context, the adjunctive use of a YAP agonist could potentially counterbalance the effects of statins, preserving the integrity of the pregnancy. However, the clinical landscape is yet bereft of a specific YAP agonist. XMU-MP-1 has been a staple in laboratory settings as a YAP agonist [161], underscoring the imperative for the development and clinical validation of novel, efficacious YAP-activating compounds.

The functional integrity of fetal membranes is crucial for maintaining a pregnancy. Fetal membranes consist of two distinct tissues: the amnion and the chorion. The amnion is believed to govern the mechanical behavior of fetal membranes. It acts as a structural barrier [162], while the chorion acts as an immune barrier, protecting the fetus from the maternal immune system and preventing degradation of the amnion [163]. Recent research underscores the pivotal role of the chorion layer, especially CTCs, in facilitating maternal–fetal communication and material exchange. Notably, the intricate regulation of endogenous calcium channel inhibitor activity and the prostaglandin E2 metabolic pathway within CTCs is instrumental in thwarting maternal uterine activation triggered by fetal-derived signals [109, 164]. However, the perturbation of progesterone levels and utero inflammation can lead to preterm premature rupture of membranes (pPROM) by impairing the functions of the chorion layer [165, 166]. It has been reported that pPROM is associated with a thin chorion layer, with an estimated 37% of cases presenting an indiscernible chorionic layer, a phenomenon linked to chorionic cell senescence and apoptosis [167, 168]. CTCs are the primary source of progesterone in fetal membranes. A decline in progesterone levels and signaling is implicated in instigating a proinflammatory state within the uterus [165, 169]. Existing literature attests to the role of progesterone in augmenting cardiomyocyte proliferation, mediated by the stimulation of YAP's transcriptional activity. This raises the imperative for extensive research to elucidate the potential influence of progesterone on the senescence and apoptosis of CTCs through YAP regulation. YAP-induced cell proliferation has been heralded for its reparative effects on barriers across various tissues and organs [170]. The attenuation of the chorion layer, observable in pPROM cases, is potentially associated with diminished YAP-mediated barrier repair mechanisms, but more direct experimental evidence is needed.

Progesterone, a steroid hormone crucial for implantation and maintenance of pregnancy, is associated with a high risk of miscarriage when present at low levels [171, 172]. Mifepristone, a progesterone receptor antagonist, is frequently administered alongside prostaglandins for the medical termination of early pregnancies [173]. Notably, the use of mifepristone within the initial 39 days of gestation has been linked to an increase in apoptotic cells within the chorionic villi [174]. The potential association between this phenomenon and YAP dysfunction warrants comprehensive investigation.

In this review, we have summarized studies highlighting the role of YAP dysfunction in trophoblast cells and its implication in the onset of pregnancy complications. A limited yet insightful number of research have also unveiled the intricate involvement of proteins that modulate YAP activity, accentuating their role in fetal membrane dysfunction. In light of these findings, we advocate for strategically employing YAP agonists or antagonists as potential therapeutic interventions to ameliorate pregnancy complications. The exigency for extensive research to ascertain the optimal timing and specific types of YAP modulators for efficacious and safe application is emphatically underscored.

## Data Availability

Not data availability.

## Abbreviations

15-PGDH: NAD + -dependent 15-hydroxyprostaglandin dehydrogenase
AJs: Adherens junctions
AMOT: Angiomotin family of proteins
ANGPTL4: Angiopoietin-like 4
APC: Adenomatous polyposis coli
CCTs: Cell column trophoblasts
CDX2: Caudal-type transcription factor
CK1: Casein kinase 1
COX2: Cyclooxygenase 2
CTBs: Cytotrophoblasts
CTCs: Chorion trophoblast cells
dCCT: Distal cell column trophoblasts
E2: 17β-Estradiol
EVTs: Extravillous trophoblasts
FGR: Fetal growth restriction
FZD: Frizzled protein
GPCRs: G protein-coupled receptors
GPER: G protein-coupled estrogen receptor
GSK3β: Glycogen synthase kinase 3β
HDAC7: Histone deacetylase 7
ICM: Inner cell mass
iEVT: Interstitial extravillous trophoblast
IL-1β: Interleukin-1β
IUGR: Intrauterine growth restriction
IκB: Inhibitor kappa B
LATS1/2: Large tumor suppressor 1/2
LEF: Lymphoid enhancer-binding factor
LPA: Lysophosphatidic acid
LPS: Lipopolysaccharide
LRP-5/6: Lipoprotein receptor-related protein 5/6
MAP4Ks: Mitogen-activated protein kinase kinase kinase kinase
MOB1A/B: MOB kinase activator
MST1/2: STE20-like protein kinase 1/2
NDR1/2: The nuclear Dbf2-related (NDR) kinases 1/2
NHERF1: Na + /H + exchanger regulatory factor 1
NICD: Notch intracellular domain
pCCT: Proximal cell column trophoblast
PE: Preeclampsia
PG: Prostaglandin
PKCζ: Protein kinase C ζ
pPROM: Preterm premature rupture of membranes
PS: Primitive Syncytium
PTPN14: Protein tyrosine phosphatase nonreceptor type 14
RBP-Jκ: Recombining binding protein suppressor of hairless
RPL: Recurrent pregnancy loss
RSA: Recurrent spontaneous abortion
S1P: Sphingosine 1-phosphate
S1PR2: Sphingosine-1-phosphate receptor-2
SAV1: Salvador homolog 1
sPE: Severe preeclampsia
STBs: Syncytiotrophoblasts
TAD: Transcription activation domain
TAK1: Transforming growth factor-beta-activated kinase 1
TCF: T-cell-specific factor
TE: Trophectoderm
TEAD: TEA Domain Transcription Factor
TJs: Tight junctions
TNFR1: Tumor necrosis factor receptor 1
TNF-α: Tumor necrosis factor-alpha
TSCs: Trophoblast stem cells
USP31: Ubiquitin-specific peptidase 31
VDD: Vitamin D deficiency
YAP: Yes-associated protein
ZO-1/2: Zonula occludens 1/2
